# Hybridizing Deep Neural Networks and Machine Learning Models for Aerial Satellite Forest Image Segmentation

**DOI:** 10.3390/jimaging10060132

**Published:** 2024-05-29

**Authors:** Clopas Kwenda, Mandlenkosi Gwetu, Jean Vincent Fonou-Dombeu

**Affiliations:** 1 School of Mathematics, Statistics and Computer Science, University of KwaZulu Natal, Pietermaritzburg 3209, South Africa; fonoudombeuj@ukzn.ac.za; 2 Department of Industrial Engineering, University of Stellenbosch, Stellenbosch 7600, South Africa; mgwetu@sun.ac.za

**Keywords:** segmentation, machine learning, supervised approach, deep learning

## Abstract

Forests play a pivotal role in mitigating climate change as well as contributing to the socio-economic activities of many countries. Therefore, it is of paramount importance to monitor forest cover. Traditional machine learning classifiers for segmenting images lack the ability to extract features such as the spatial relationship between pixels and texture, resulting in subpar segmentation results when used alone. To address this limitation, this study proposed a novel hybrid approach that combines deep neural networks and machine learning algorithms to segment an aerial satellite image into forest and non-forest regions. Aerial satellite forest image features were first extracted by two deep neural network models, namely, VGG16 and ResNet50. The resulting features are subsequently used by five machine learning classifiers including Random Forest (RF), Linear Support Vector Machines (LSVM), k-nearest neighbor (kNN), Linear Discriminant Analysis (LDA), and Gaussian Naive Bayes (GNB) to perform the final segmentation. The aerial satellite forest images were obtained from a deep globe challenge dataset. The performance of the proposed model was evaluated using metrics such as Accuracy, Jaccard score index, and Root Mean Square Error (RMSE). The experimental results revealed that the RF model achieved the best segmentation results with accuracy, Jaccard score, and RMSE of 94%, 0.913 and 0.245, respectively; followed by LSVM with accuracy, Jaccard score and RMSE of 89%, 0.876, 0.332, respectively. The LDA took the third position with accuracy, Jaccard score, and RMSE of 88%, 0.834, and 0.351, respectively, followed by GNB with accuracy, Jaccard score, and RMSE of 88%, 0.837, and 0.353, respectively. The kNN occupied the last position with accuracy, Jaccard score, and RMSE of 83%, 0.790, and 0.408, respectively. The experimental results also revealed that the proposed model has significantly improved the performance of the RF, LSVM, LDA, GNB and kNN models, compared to their performance when used to segment the images alone. Furthermore, the results showed that the proposed model outperformed other models from related studies, thereby, attesting its superior segmentation capability.

## 1. Introduction

Forest constitute a greater portion of the natural ecosystem and is one of the richest forms of resource that contributes to the Gross National Product (GNP) of many nationalities. They play a pivotal role in areas such as climate regulation, environmental improvement, the global water cycle, and soil conservation [1,2]. Apart from a wider range of ecological services, forests contribute to socio-economic through the provision of forest product such as timber and also offers nature-based recreation [3]. Therefore it is of paramount importance to continuously monitor forests in order to understand the changes that occur with respect to time. Surveys were used to conduct forest monitoring, but such a technique is costly and cannot be completed in a short period of time [4]. With the development of advanced modern sensors, remote sensing has made it possible to monitor land cover on a large scale. However, the automatic segmentation of aerial satellite images to visualize areas that are populated with forests remains a challenging task [5]. In fact, the process of segmenting images of remote sensing of the Earth’s surface has not been brought to automation with the same accuracy as with manual marking [6], despite the rapid development of computer vision algorithms for detecting objects in an image. Although humans can outperform computers in solving segmentation problems, doing so manually would take too long. Satellite image segmentation using computer vision algorithms is a very pertinent task in this scenario because it is hard to obtain segmentation results in real time. The segmentation process is generally a difficult task due to two main challenges: one is the intrinsic ambiguity in image perception and the other is that an image with many visual patterns becomes too complex to model [7].

The field of computer vision has witnessed remarkable advancement, largely propelled by the development of deep learning techniques. Deep neural networks such as VGG16 and ResNet50 have emerged as powerful tools for extracting intricate features from images, enabling tasks such as object detection, and image classification and segmentation. The recent development of deep neural networks such as Convolutional Neural Networks (CNN) has improved images segmentation results [8,9]. However, deep-learning techniques produce excellent results when trained on large data sets, which is contrary to traditional machine-learning techniques which produce good results on a limited dataset. On the other hand, traditional machine learning classifiers for segmenting images such as Linear Support Vector Machines (LinearSVM), k-nearest neighbor (kNN), Linear Discriminant Analysis (LDA), and Gaussian Naive Bayes (GNB), lack the ability to extract features such as the spatial relationship between pixels and texture, resulting in subpar segmentation results when used alone. This study aims to address this shortcoming of traditional machine learning algorithms by proposing a hybrid model that combines the strengths of deep neural networks and traditional machine learning algorithms for improved segmentation of aerial satellite images. Features of aerial satellite images are firstly extracted with VGG16 and ResNet50 deep neural network models. The resulting features are subsequently used by RF, LinearSVM, kNN, LDA, and GNB classifiers to perform the final segmentation. VGG16 and ResNet50 were chosen in this study because they have innately dissimilar network architectures that abstract unrelated information for the purpose of object detection. The performance of the proposed hybrid model was evaluated using various metrics including Accuracy, Jaccard score index and Root Mean Square Error (RMSE). The aerial satellite forest images were obtained from a deep globe challenge dataset. The performance of the proposed model was evaluated using various metrics such as Accuracy, Jaccard score index and Root Mean Square Error (RMSE). The experimental results demonstrated that the RF model outperformed other classifiers in the hybrid approach, achieving best segmentation results with an accuracy of 94%, a Jaccard score of 0.913, and an RMSE of 0.25. The experimental results also revealed that the proposed model has significantly improved the performance of the RF, LSVM, LDA, GNB, and kNN models, compared to their performance when used to segment the images alone. Furthermore, the results showed that the proposed model outperformed other models from related studies, thereby, attesting its superior segmentation capability. The Contribution of the paper is summarised as follows:

- We introduce a novel image segmentation approach that leverages an ensemble of ResNet50 and VGG16 models. In this approach, features extracted from both ResNet50 and VGG16 are combined to generate a comprehensive set of features, encompassing all possible information. These integrated features are then utilized by machine learning classifiers for subsequent classification tasks.
- The novel hybrid model combines the strength of deep neural networks (ResNet50 and VGG16 for feature extraction) and traditional machine learning classifiers for improved segmentation of aerial satellite images. Each classifier brings its unique strengths to the segmentation task, encompassing diverse methodologies and learning paradigms.

The rest of the paper is structured as follows. Section 2 reviews related studies. An overview of feature extraction algorithms is provided in Section 3. Machine learning classifiers are discussed in Section 4. Section 5 looks at the segmentation process by RF. The structure of the proposed model is presented in Section 6. Section 7 discusses the results obtained and Section 8 concludes the paper.

## 2. Related Studies

Segmenting forest, land cover and satellite images have been of interest to many researchers [10,11,12,13] in recent years. A study [10] proposed a U-net model to perform segmentation tasks on forest and water bodies satellite images. The purpose of the model was to determine the area covered by forest and water. The approach performed well as it attained a validation accuracy of 82.92% and 82.5% to perform segmentation on areas covered by water and forest respectively. This study had the challenge of having mislabelled masks in its data set. The presence of mislabelled masks hugely contributes to the decrease in model performance. The removal of mislabeled masks undoubtedly contributes to improved model performance, it is essential to acknowledge the potential limitations of this approach. Data cleaning processes, though necessary, can be time-consuming and resource-intensive, particularly for large datasets. Moreover, the effectiveness of data preprocessing techniques may vary depending on the specific characteristics of the dataset and the nature of the segmentation task. In this proposed study, the data set used did not contain any mislabelled mask. The cleaning process of removing the mislabelled mask is very essential prepossessing task, as this has got a bearing on the overall model performance. The absence of mislabelled masks in the proposed study offers a distinct advantage over the previous work, as it eliminates a significant source of error that could compromise the accuracy of segmentation results. By employing clean, accurately labeled data, researchers can mitigate the risk of introducing biases or inaccuracies into the model training process, thereby enhancing the robustness and generalisability of the segmentation model.

Another study [11] came up with a model based on U-net adopted under transfer learning to perform agricultural field segmentation on satellite imagery. The model integrated the strength of transfer learning, residual network, and U-net architecture (TL-ResUnet). The approach was tested on satellite images obtained from the DeepGlobe data set. The model outperformed other methods such as DFCNet, DeepLabv3, and DeepLabv3+ in terms of Intersection over Union (IoU). The TL-ResUnet obtained an IoU of 81%, DeepLabv3 (74.5%), and DeepLabv3+ (75.6%). However, in terms of robustness, the model failed in some circumstances to segment small forested areas and narrow water bodies because of the presence of noise in satellite images. To overcome this challenge the proposed study used a non-local means algorithm to denoise the input images. This approach demonstrates the importance of addressing image noise in satellite imagery to improve segmentation accuracy and robustness. The first study emphasizes the importance of data quality and preprocessing, while the second study highlights the need to mitigate noise in satellite images for accurate segmentation. Synthesizing these insights, it becomes evident that advancements in satellite image segmentation require a multifaceted approach, incorporating techniques from computer vision, deep learning, and image processing. Furthermore, addressing specific challenges such as data quality and image noise is crucial for developing robust and reliable segmentation models capable of accurately delineating various environmental features from satellite imagery.

Authors in [12] used different machine learning algorithms such as Fully convolutional neural network (FCNN), linear support vector machine, naive bayes, and logistic regression to perform semantic segmentation on satellite images to determine the allotment of forested areas in order to determine the rate at which deforestation occurs over a period of time. The FCNN achieved the highest Jaccard index score of 91.8% followed by the regression logistics with 90%. However, because of a huge imbalance in the data set the model did not perform well in detecting non-forest areas. Another challenge for FCNN was that it required a lot of training time and it consumed a significant amount of storage space.

Segmentation of forested regions in aerial images was accomplished in a study [6] using an Unet network model with 2 encoders. The model was applied to a dataset consisting of 17 images, each of which had a 16-bit channel. With a dice coefficient of 0.765, the model demonstrated its ability to segment forests in satellite images. Due to experts’ inability to completely segment ground truth images, the model’s detection performance was subpar, with an F-measure of 0.349. This proposed research uses a fully segmented ground truth image to overcome this challenge. By ensuring that the ground truth data accurately represents the forested regions, the researchers seek to provide a more reliable basis for training and evaluating the segmentation model. The study by [6] and the proposed research in this study reveal common themes and divergent approaches in addressing the segmentation of forested regions. Both studies utilize deep learning networks, indicating their effectiveness in image segmentation tasks. However, they encounter different challenges: the first study struggles with incomplete ground truth annotations, while the proposed research aims to mitigate this issue by utilizing fully segmented ground truth images. These perspectives highlight the importance of accurate and comprehensive ground truth data in training and evaluating segmentation models. While achieving high-performance metrics like the dice coefficient is indicative of good segmentation quality, the reliability of these metrics hinges on the quality of the ground truth annotations.

Another Model based on Unet was developed to perform the segmentation of deforestation areas using satellite images taken from Ukrainian forests [14]. Satellite images at a resolution of 512 by 512 pixels contains sections of forest, deforestration, and other areas. The dataset had an imbalance issue, however, the hybrid loss function was employed to overcome the challenge. To evaluate the effectiveness of the model as well as its consistency during the process of validation and training, k-cross validation and random runs were used. The model had an intersection over union (IOU) mean of 0.03 and an intersection over union standard deviation (IOU std) of 0.03 after running it for 100 epochs. According to these findings, the unpredictability of the initialization process and the variety of the photos did not have a major impact on the performance of the model. However, wider data variability decreased model’s performance.

A study in [15] developed a supervised artificial neural network for plant image segmentation using a raw image dataset of 8-bit RGB intensity values. The neural network structure is composed of 1024 neurons in the first hidden layer, then 512 neurons in the second layer. The ReLU activation function is employed between the input layer and the first hidden layer. The sigmoid function is used between the hidden layer and the output layer. The output layer has one neuron used to compute an instance belonging to a class. The model performed well as it produced a very low error rate of 0.007. However, the approach took a significantly huge amount of time to segment images of high resolution, therefore, the study recommended using distributed computing or a graphical processing unit (GPU) to speed up the segmentation time. It is, for this reason, the proposed model in this study adopted the cloud-based GPU platform for implementation. In response to the computational challenges identified in the prior study, the proposed model in this study leverages a cloud-based GPU platform for implementation. This approach aims to harness the parallel processing capabilities of GPUs to accelerate image segmentation and reduce processing time. By offloading computation to a cloud-based GPU infrastructure, the proposed model seeks to overcome the computational bottleneck associated with high-resolution image segmentation. The neural network architecture described in [15] achieves impressive segmentation performance but at the cost of prolonged processing times, particularly for large datasets or high-resolution images. In contrast, the adoption of cloud-based GPU acceleration offers a pragmatic solution to enhance computational efficiency without compromising segmentation accuracy. By harnessing the parallel processing power of GPUs through cloud infrastructure, the proposed model aims to streamline the segmentation process and improve scalability for real-world applications.

Another study [16] employed the U-Net Neural Network with ResNet34 to conduct wildfire segmentation on satellite pictures. The adaptive moment estimation approach was utilized to achieve optimal results during the training of the model. The Resurs dataset which is made up of 10-bit images with three channels that have a spatial resolution of between 1 and 10 m per pixel, and the Planet dataset, which is made up of 10-bit satellite images with three channels that have a spatial resolution of 3 m per pixel were utilized. The performance of the model was satisfactory, as it obtained a Jaccard score index of 0.87 for the Resurs dataset and 0.757 for the planet dataset. However, the application of random chromatic distortion to boost the model’s robustness in the face of noisy images resulted in a minor decline in the quality of the deep learning method. A study in [16] employs deep learning techniques, specifically U-Net with ResNet34, highlighting the efficacy of deep neural networks for image segmentation tasks across various domains. Additionally, the use of specific optimization techniques, such as adaptive moment estimation, underscores the importance of algorithmic optimization for improving model performance. The study also introduces a novel challenge related to image preprocessing. While attempting to enhance model robustness with random chromatic distortion, the study inadvertently observes a slight decrease in segmentation quality. This highlights the intricate balance between data augmentation techniques and model performance, underscoring the need for careful consideration of preprocessing methods in deep learning pipelines.

Drones, with their excellent spatial resolution and adaptability in picture capture, have just ushered in a new era in the mapping of wetlands. A study in [17] used machine learning algorithms and deep learning algorithms using drone imagery to make a map of the important plant groups in Clara Bog, an Irish wetland, before spring. The highest accuracy in semantic segmentation (about 90%) was achieved by combining the ResNet50 and SegNet architectures, and the Random forest (RF) was found to be the best pixel-based machine learning classifier. When used with the graph cut method for image segmentation, it gave good accuracy of 85%. However, the deep learning architecture’s main challenge is computational overhead. To address this issue, the proposed model in this study has adopted an ensemble of VGG16 and ResNet50 models solely for feature extraction and the segmentation process is then performed by the Random Forest algorithm. Comparing these approaches underscores the trade-offs between deep learning and machine learning techniques in wetland mapping. Deep learning excels in extracting complex features from drone imagery, enabling high-level semantic segmentation with impressive accuracy. However, this comes at the cost of computational resources and processing time, particularly for large datasets. On the other hand, machine learning algorithms like Random Forest offer computational efficiency and interpretability, making them well-suited for segmentation tasks. By integrating deep learning for feature extraction and machine learning for segmentation, the proposed ensemble model seeks to strike a balance between accuracy and computational efficiency.

Ref. [13] implemented a random forest algorithm on SPOT satellite imagery to identify the best segmentation scales to predict land cover classes. The algorithm achieved an overall accuracy of 85.2%. The study used the Normalised Difference Vegetation Index (NDVI) and Normalised Difference Water Index (NDWI) to extract features. However, NDVI is affected by saturation, atmosphere effects, and sensor quality [18]. It is, for this reason, the proposed study adopts the hybrid approach of deep learning networks which excels at extracting features regardless of atmospheric effects. A study in [19] employed convolutional neural networks (CNN) to segment an aerial satellite image into different regions, but the study encountered challenges of having isolated satellite and segmentation images made at different times leading to inaccuracy. Another CNN segmentation model in [20] was employed to detect forest fires in aerial satellite images. Sai et al. [21] developed a model that used NDVI to separate forest and dense grass in satellite vegetation images. Using only spectral characteristics to distinguish grass areas from forests may not yield greater accuracy, however, deploying machine-learning algorithms would yield greater accuracy for complex features.

## 3. Overview of Feature Extraction Algorithms

In this study, ResNet50 and VGG16 deep learning models were used to extract features based on the studies done by [22,23]. The salient features of the models are described as follows:

### 3.1. VGG16 Network Model

The VGG network model was first proposed by the Visual Geometric Group at Oxford University, and that is where its name was derived. The network became much more popular in 2014 when it won first and second place in the classification and localization task when it participated in the ImageNet Large Scale Recognition Challenge (ILSVRC) [24]. The network is composed of 13 convolutional layers and 3 fully connected layers, hence the name VGG16. The large convolution filter in the VGG16 network has been replaced by various 3 × 3 convolutional filters stacked on top of each other. The multiple 3 × 3 convolutional filters make the network deeper and at the same time reduce the number of total parameters [25]. In the VGG16 network, each max-pooling layer has a kernel of size 2 and a step of 2.

### 3.2. ResNet50 Network Model

**Figure 1:**
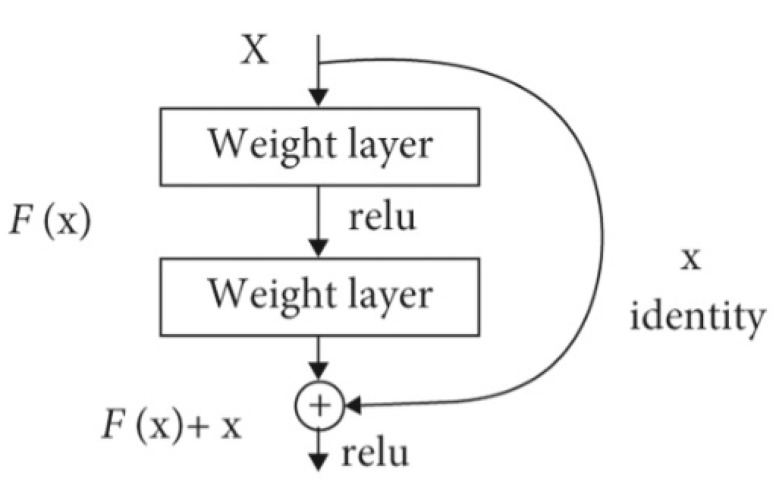
ResNet50 shortcut.

ResNet50 is a 50-layer-deep CNN and is the first network to adopt residual learning in 2015 [26]. The network won the first prize in 2015 when participated in computer vision benchmarking challenges in the ILSVRC and Microsoft Common Objects in Context2015. A deep layered network suffers from increased error rates due to the vanishing gradient problem [27]. However, the ResNet50 models solve this challenge by incorporating a technique called skip connections or shortcuts as shown in Figure 1. The shortcuts jump several layers and connect directly to the input, hence the vanishing gradient is avoided. The mapping function of a shortcut connection sums up the input instance and the output instance such that the original mapping function

(1) H(x)=F(x)−x

is redefined as

(2) H(x)=F(x)+x

The refinement of the mapping function makes learning simple whilst the desired functionality is achieved. The mapping function presented in Equation (2) is implemented through feed-forward neural networks as presented in Figure 1.

## 4. Overview of Machine Learning Classifiers

### 4.1. Random Forest Algorithm

The random forest algorithm is an ensemble classifier based on decision trees, where each tree grows through randomization. The RF algorithm is capable of processing large amounts of data at high speed using decision trees. During the training phase, the RF algorithm randomly chose a subset of data from the training data. At a particular node, say *n*, the training data is recursively split into left and right subsets using the split function and the threshold. The split function randomly selects the threshold in the range $h\in (minX\left(v_{i}\right),maxX\left(v_{i}\right)$ where h is the threshold and $X(v_{i})$ is the split function of vector *v*. The split function that creates the left and right subset trees is expressed as:

(3) $$m_{l}=\left(i\in m_{n}\right)\left|x\left(v_{i}\right)<h\right)$$

(4) $$m_{r}=m_{n}\setminus m_{i}$$

where $m_{l}$ is left data, $m_{r}$ is right data and $m_{n}$ is data at corresponding node *n*. At the split node, several candidates are randomly produced through the split function and the threshold. Only candidates that maximize the information gain at a given node are selected. The information gain is computed by entropy estimation as expressed in Equation (11).

(5) $$\Delta E=-\frac{|m_{i}|}{|m_{n}|}E\left(m_{n}\right)-\frac{|m_{r}|}{|m_{n}|}E\left(m_{n}\right)$$

where ΔE is the information gain. Whenever the training process reaches a leaf node or no more ΔE is possible, the iterative process stops. The final class is generated by the ensemble of all distributed trees X = $\left(x_{1},x_{2},\ldots ,x_{n}\right)$ as presented in Equation (16):

(6) $$P\left(c_{i}|X\right)=\frac{1}{N}\sum_{n=1}^{N}P\left(c_{i}|x_{N}\right)$$

where $P(c_{i}|X)$ is the probability of class $c_{i}$ given distributed trees X.

### 4.2. Linear Support Vector Machines

Linear Support Vector Machines (LinearSVM) is a machine learning classification technique that was proposed by Vapnick and his group at AT&T BELL laboratories [28,29]. LinearSVM works on obtaining the best generalization performance by ensuring a relationship balance between accuracy obtained from the training data and the machine capacity. It works by trying to separate classes with a hyperplane surface so as to maximize the margin among them. LinearSVM has also been successfully applied to perform handwritten digit recognition, face detection on images, and object detection [30]. Based on Vapnick, LinearSVM can either be described either from the linearly separable case or Non-linearly separable case.

#### 4.2.1. Linearly Seperable Case

For this case, data is considered to be linearly separable, and the plane is defined by an equation: v·x+c=0, where *x* is a specific point on a hyperplane, and *v* is an m-dimensional vector that is perpendicular to the hyperplane, and *c* is the distance of the point is closest to the hyperplane origin. This will arise two inequality equations:

(7) $$v\cdot x_{i}+c\geq 1,\;\;for\;\;y_{1}=+1,\;\;and$$

(8) $$v\cdot x_{i}+c\leq 1,\;\;for\;\;y_{1}=-1$$

Equations (7) and (8) can be combined into

(9) $$y_{i}\left(v.x_{i}+c\right)-1\geq 0,\forall i$$

Now LinerSVM will try to find a hyperplane v·x+c=0 with minimum ${\left||v|\right|}^{2}$. This is also equivalent to determining the hyperplane with the largest margin, which is determined by calculating the distance between the closest vectors for two classes. Hence the problem is redefined as follows:

(10) $$Minimize\;\underset{︸}{v,c}\;\frac{1}{2}{\left||v|\right|}^{2}\;\;subject\;\;to\;\;y_{i}\left(v\cdot x_{i}+c\right)-1\geq 0$$

#### 4.2.2. Non-Linear Case

For this case, data appears in the optimization problem in the form of dot products. It maps feature vectors to a higher dimensional Euclidean space by a mapping:

(11) $$\Phi :R^{d}\alpha H$$

Then the optimization problem in space *L* is obtained by replacing $x_{i}\cdot x_{j}$ by $\Phi \left(x_{i}\right)\cdot \Phi \left(x_{j}\right)$. If there is kernel function *Q* defined by

(12) $$Q\left(x_{i},x_{j}\right)=\Phi \left(x_{i}\right)\cdot \Phi \left(x_{j}\right)$$

then there is only a need to compute $Q(x_{i},x_{j})$ in the training maps. The decision function then becomes

(13) $$f\left(x\right)=sign(\sum_{i=1}^{I}y_{i}\lambda_{i}Q\left(x,x_{j}\right)+c)$$

### 4.3. k Nearest Neighbor

The K nearest neighbor algorithm is another machine learning technique that is employed for regression and classification-related tasks. It works by assigning unmarked data points to the class that is nearest to the labeled data point [31]. The algorithm’s efficacy is through its ability to leverage similarity metrics, which consider the distance between points to determine the most analogous data point. K-nearest neighbor applies information obtained from the observed data to make its predictions rather than relying on predefined associations between the predictor and predicted variable. Fore regression related tasks, the K-NN approximates the response of a test point $\left(x_{p}\right)$ by considering the weighted average of all the responses from the closest point (x(1),x(2),…,x(k)) in the vicinity of $\left(x_{p}\right)$. In order to determine the right weight to assign to each neighbor; kNN adopts a kernel function that calculates the weight of the neighbor based on its proximity to the test point. For a given training dataset $X=\{x_{1},x_{1},\ldots ,x_{s}\}$ consisting of s training points, each with *T* features, weighted Euclidean distance can be employed to determine the distance between each training point $x_{i}$ and the test point $\left(x_{p}\right)$. Euclidean Distance (ED) is computed as presented in the Equation (14)

(14) $$ED\left(x_{p},x_{i}\right)=\sqrt{\sum_{t}^{T}w_{t}{\left(x_{p,t}-x_{i,t}\right)}^{2}}$$

where *T* represents the number of features, $x_{p,t}$ denotes the *t*th feature value of the existing point $x_{p}$, $x_{i,t}$ denotes the *t*th feature value of training point $x_{i}$. The *t*th feature weight is represented by $w_{t}$. The kernel regression that is used to estimate the response of $x_{p}$ is defined in Equation (15)

(15) $$f\left(x_{p}\right)=\frac{\sum_{i=1}^{k}\phi \left(x_{p},x_{i}\right)f\left(x_{i}\right)}{\sum_{i=1}^{k}\phi \left(x_{p},x_{i}\right)}$$

where *k* is the number of k-nearest neighbors, $\phi \left(x_{p},x_{i}\right)f\left(x_{i}\right)$ is the kernel function at the *i*th training point and $f(x_{i})$ is the known response of $x_{i}$.

### 4.4. Linear Discriminant Analysis

The Linear Discriminant Analysis is employed to decide to differentiate between input patterns [32]. For a given two classes the decision boundary is defined as:

(16) $$d\left(Q\right)=Q_{1}-mQ_{2}-r$$

where $Q_{1}$ and $Q_{2}$ represents input patterns. The idea behind LDA is to construct a decision surface such that d(Q)>0 would categorize patterns for one class and d(Q)<0 would categorize patterns for another class. Considering that $x=\{x_{1},x_{2},\ldots ,x_{M}\}$ is an *M* dimensional pattern vector. Suppose the number of classes is *n* and the problem is to classify a given instance *x* to any one of the classes. The problem is solved by defining *n* decision functions given by $d_{1}x,d_{2}x,\ldots ,d_{n}x$. The instance x would be categorized into class *p* and not *q* if

(17) $$d_{p}\left(x\right)>d_{q}\left(x\right)\;\;where\;p\neq q\;\;if\;\;p,q=1,2,3,\ldots ,n$$

The decision boundary between the two classes *p* and *q* will be redefined as

(18) $$d_{p}\left(x\right)-d_{q}\left(x\right)=0$$

Therefore the instance x would be classified into class p if

(19) $$(d_{p}\left(x\right)-d_{q}\left(x\right))>0$$

and to class *q* if

(20) $$(d_{p}\left(x\right)-d_{q}\left(x\right))<0$$

### 4.5. Gaussian Naive Bayes

Gaussian Naive Bayes simplifies learning by assuming that features are independent of given classes [33]. This assumption is described by many researchers as poor in general, but however, it works effectively based on this assumption. The Bayesian Classifier assigns a given instance *x* to the most likely class as expressed in the Equation (21)

(21) $$P\left(C\right)=\prod_{i}^{n}=Ip\left(X_{i}|c\right)$$

where *C* denotes the classifier, and $X=(X_{1},\ldots ,X_{n})$ represents a feature vector [34]. The Gaussian Naive Bayes is a simplified version of Bayesian probability based on the independence assumption. This implies that one attribute of the probability of one has no impact on the probabilities of the other attributes.

## 5. Evaluation Metrics Used in the Study

Metrics such as the Jaccard index, Root Mean Square Error (RMSE), confusion matrix, ROC_−_AUC curves, Precision, Recall, F1-Score, and Accuracy are used in this study to evaluate the performance of the proposed segmentation model. The confusion matrix facilitates the visualization of the model’s performance. The visualization platform makes it simple to identify confusion between regions, e.g., it is simple to determine which regions have more misclassified pixels than others. The ROC_−_AUC curve, also known as the sensitivity measure, is a graph of the true-positive rate versus the false-positive rate. Better classification performance is indicated by a model with a trajectory that is located far from the median. In a plot, the ROC_−_AUC curve represents the efficacy of a model across all thresholds. The bigger the area, the better the model. One of the benefits of the ROC_−_AUC curve is that it facilitates the comparison of results from various models without the need to reconcile sensitivity and specificity concerns. The ROC_−_AUC formula for binary classification is expressed in Equation (22) [35].

(22) $$ROC_{-}AUC=\frac{x_{p}-m_{p}\left(m_{p}+1\right)/2}{m_{p}m_{m}}$$

where $x_{p}$ denotes the sum of all positive ranked samples. $m_{p}$ and $m_{m}$ represent the number of negative and positive samples, respectively.

The Jaccard index, also referred to as the Intersection-Over-Union (IoU) is the widely used metric for evaluating the predictions of segmentation models. IoU is defined by the area of overlap between the predicted segmented image and the reference image(ground truth) divided by the union area of the segmented image and the reference image. The IoU is defined in Equation (23).

(23) $$IoU=\frac{TP}{TP+FP+FN}$$

where TP denotes true positive, TN represents true negative, FN represents false nagative and FP denotes false positive. Accuracy determines the efficiency of the model by considering the total correct predictions made by the segmentation method. Accuracy is expressed in Equation (24).

(24) $$Accuracy=\frac{TP+TN}{TP+TN+FP+FN}$$

The root-mean-square error RMSE is the square root of the mean square of all errors. Because it is scale-dependent, RMSE is a good measure of accuracy for comparing forecasting errors of different models or model configurations for a specific variable but not between variables. It is calculated in Equation (25).   

(25) $$RMSE=\sqrt{\frac{1}{n}\sum_{i=1}^{n}{\left(O_{i}-P_{i}\right)}^{2}}$$

where $O_{i}$ are the actual values and $P_{i}$ are the predicted value Recall also referred to as sensitivity is a measure of instances predicted as positive against all actual positive values. This metric returns the fraction of positive patterns that are correctly classified. Recall metric is computed by Equation (26)

(26) $$Recall=\frac{TP}{TP+FN}$$

Precision returns the proportion or fraction of positive identification (true positives) that were correct. Precision is expressed in Equation (27).

(27) $$Precision=\frac{TP}{TP+FP}$$

F1-Score is the harmonic average between precision and recall rates. This metric is expressed in Equation (28)

(28) $$F1-Score=2\times \frac{Precision\times Recall}{Precision+Recall}$$

## 6. Materials and Methods

### 6.1. Data Set

**Figure 2:**
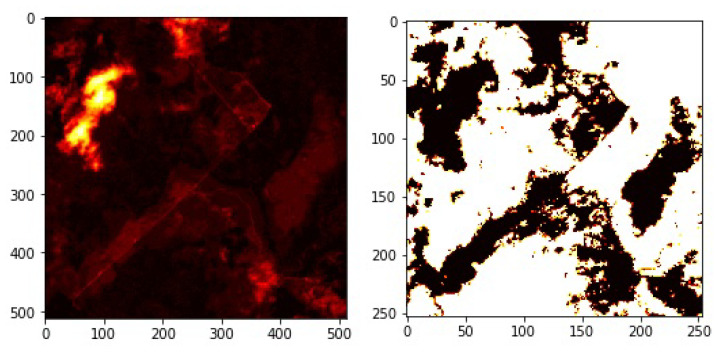
The right panel shows the extracted RGB patches and the left panel shows its corresponding masks.

The aerial image used for the study was obtained from the Land Cover Classification Truck in the DeepGlobe Challenge data set [36]. The associated reference image in the data set is binary in nature, it only shows forest region areas and non-forest region areas. Figure 2 shows an original image and its corresponding labeled mask from the dataset.

### 6.2. Experimental Setup

Deep neural networks such as VGG16 and ResNet50 require substantial resources, especially during the training phase hence requiring high-performance GPU (Google Processing Unit) and TPU (Tensor Processing Units). Therefore the experiment was conducted on the Google Colab environment which provides free GPU and TPU cloud resources. In particular, the experiment used GPU with the acceleration of NVIDIA Tesla due to the high computational requirements of the experiment. Table 1 Shows the hardware and software specifications for the experiments.

### 6.3. The Proposed Model

**Figure 3:**
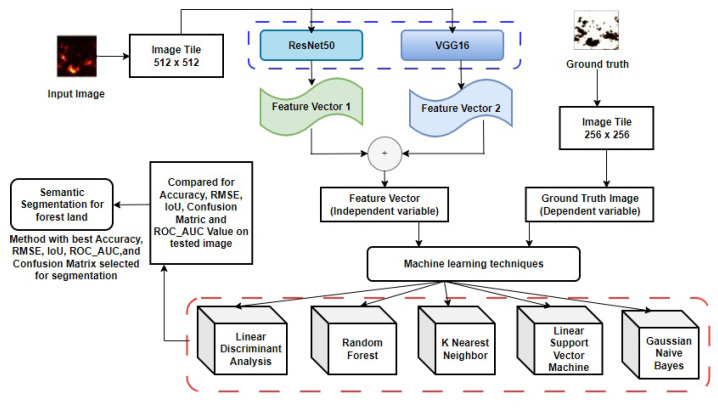
Segmentation framework model with all traditional machine learning classifiers.

The study proposes an aerial forest image segmentation model that uses a hybrid approach of ResNet50 and VGG16 deep learning models to generate a set of features for the machine learning algorithms to perform the segmentation process. This study chose these two pre-existing models due to their innately dissimilar architecture that abstracts unrelated information from images used for object detection purposes [37]. ResNet50, known for its residual connections, enables the effective capture of hierarchical features through its deep layer architecture [35]. This characteristic is particularly advantageous for capturing intricate details and patterns in aerial forest images, enhancing the model’s ability to discriminate between different forest elements and background noise. On the other hand, VGG16, with its simpler architecture comprising stacked convolutional and pooling layers, offers a complementary approach to feature extraction [35]. Despite its comparatively shallower depth, VGG16 excels at capturing basic image features and spatial relationships, which are crucial for delineating forest boundaries and structures. By combining the strengths of ResNet50 and VGG16, the proposed model leverages a diverse range of feature representations, enabling more robust and accurate segmentation of forested areas. The hybrid approach helps in expanding the feature vector scope. A single feature selection method only chooses the best subset of features from the training dataset. As a result, the end feature vector may not be a true reflection of the training dataset and may not be a good starting point for the next step, which is to segment the image. When different methods’ results are put together, the result may be more accurate. Features produced by the hybrid approach of deep learning models were applied to various traditional machine learning techniques such as K Nearest neighbor, Random Forest, Linear Discriminant Analysis, Gaussian Naive Bayes, and Linear Support Vector Machines to evaluate their segmenting power on aerial satellite forest test image. The general framework of the model is shown in Figure 3.

### 6.4. Validation of Algorithms

Replication is a cornerstone of scientific inquiry, serving as a critical tool for validating algorithms used and ensuring the reliability of experimental results [38]. When an experiment is replicated, it essentially involves conducting the same procedures and analyses multiple times using the same dataset or under similar conditions. The goal is to verify whether the initial findings can be consistently reproduced. In the context of this study, the same experiment was repeated several times over the same data and yielded consistent results, this underscores the robustness and reliability of the original findings. Consistency across replications provides strong evidence supporting the validity of the conclusions drawn from the experiment.

#### 6.4.1. Feature Generation

**Figure 4:**
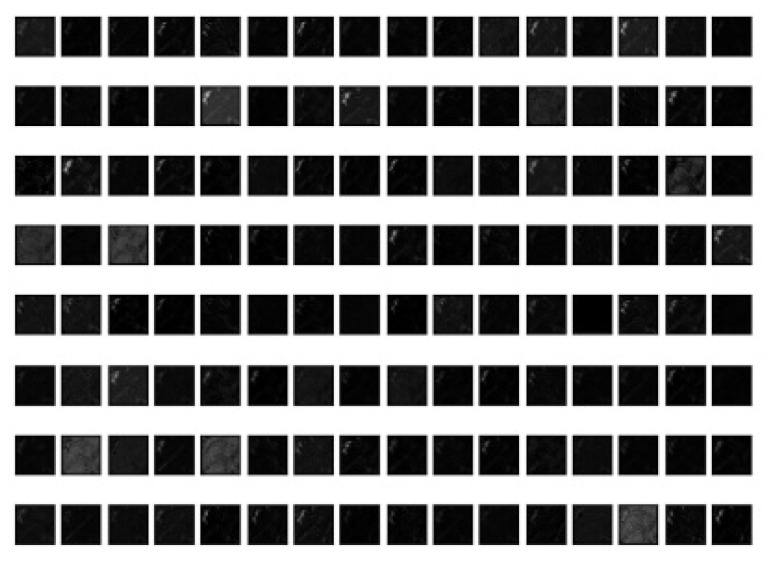
Set of features produced by VGG16 model.

**Figure 5:**
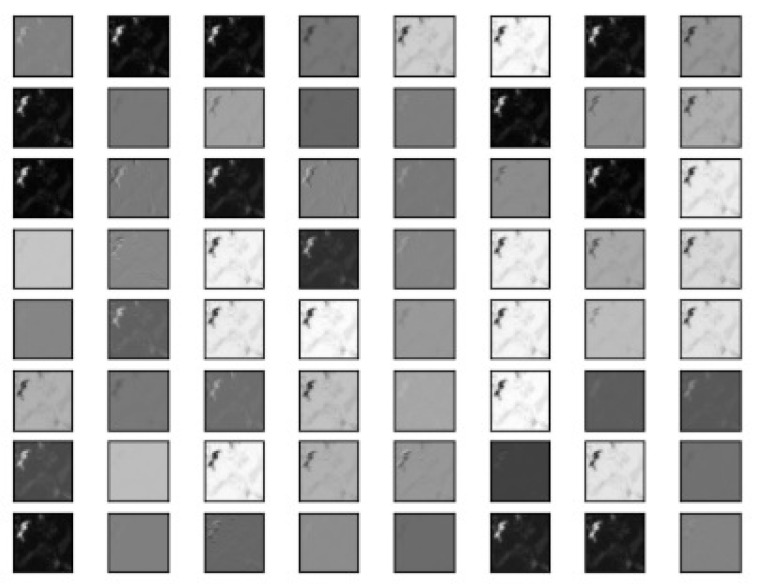
Set of features produced by ResNet50 model.

**Figure 6:**
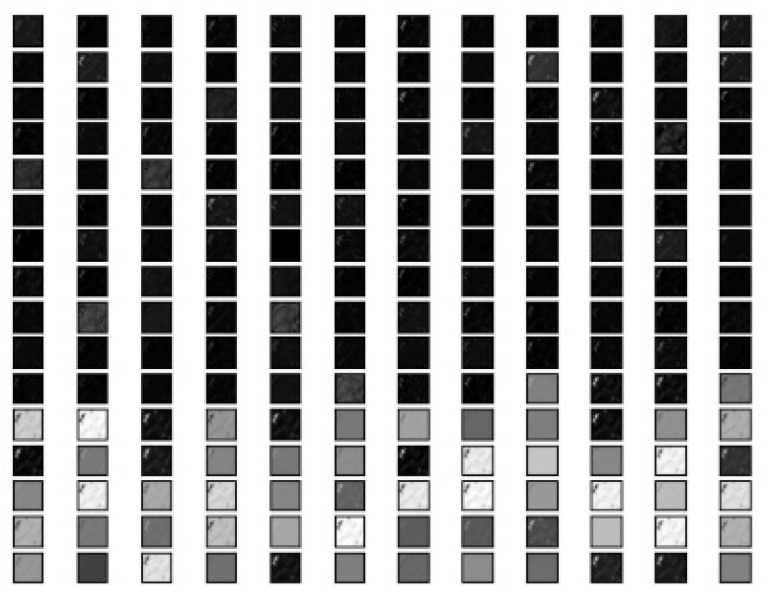
Composite set of features produced by VGG16 and ResNet50.

The VGG16 and ResNet50 deep-learning models were used to generate a set of features which were in turn used to segment an aerial forest image. VGG16 managed to produce 128 features (Figure 4) and ResNet50 managed to produce 64 features (Figure 5). The output features from both models were concatenated together to produce the final feature vector with 192 features (Figure 6).

#### 6.4.2. Segmentation Process by Machine Learning Classifiers

Combined features obtained from the hybridized approach of VGG16 and ResNet50 models are set as a dependent variable in the data frame. The pixel values obtained from the ground truth image are also set as an independent variable in the data frame. As presented in Algorithm 1, X is a vector that contains all the features extracted by VGG16 and ResNet50. These sets of features are set as independent variables. Variable M contains ground truth image pixel values which in turn are set as dependent variables. These two sets are split into a train set and a test set and the machine learning classifier is adopted to predict the segmented image.
**Algorithm 1** An algorithm for machine learning classifier segmentation 1:Input: P(y): Ground_−_truth_−_pixel_−_values 2:Input: P(x): Independent_−_variables_−_pixel_−_values 3:**for** P(x) = 0 **do** ▹ All features generated must match how features are generated for training 4:    feature1←VGG16 5:    feature2←ResNet50 6:    $feature3\leftarrow ground_{-}truth_{-}pixel_{-}values$ 7:**end for** 8:$X\leftarrow \sum_{1}^{2}feature\left(x\right)$      ▹ Features are added to the data frame 9:M←feature3          ▹ M denotes an independent variable10:X ⫫ M11:Input: Data: Train_−_set ▹ New Train Set from the extracted feature now to be loaded to classifier12:Data = train + test        ▹ Test data for accuracy testing13:model = MachinelearningClassifier()14:model.fit(X_−_train, M_−_train)15:prediction_−_test = model.predict(X_−_test)16:loaded_−_model = pickle.load(open(filename, ’rb’))    ▹ Applying trained model to segment other images17:**Return** segmented_−_image

## 7. Results and Discussion

As explained in the proposed model, the set of features generated by a hybrid approach of VGG16 and ResNet50 models is provided as an input to various machine learning classifiers. The goal is to determine the type of classifier that produces a satisfactory result. In the following subsections, the performance of these models was evaluated in terms of F1-score, precision, and recall for detecting forest areas and non-forest areas.

### 7.1. Evaluation of Machine Learning Models in Detecting Forest Region Areas

Table 2 presents the performance of each machine learning classifier in detecting forest regions in terms of F1 score, precision, and recall. The Random Forest algorithm attained the highest precision of 0.94, followed by LinearSVM, LDA, GNB, and kNN with precision scores of 0.92, 0.88, 0.86, and 0.82. On the other hand, GNB, obtained the highest recall of 0.98, outperforming the other machine-learning classifiers. Again, the RF algorithm recorded the highest f1-score compared to the other algorithms.

### 7.2. Evaluation of Machine Learning Models in Detecting Non-Forest Region Areas

Table 3 presents the performance of each machine learning classifier in detecting non-forest regions using the same metrics of F1 score, precision, and recall. The Linear Discriminant Analysis technique attained the highest precision of 0.94, followed by RF, LinearSVM, LDA, and kNN with precision scores of 0.93, 0.90, 0.88, and 0.87. On the other hand, RF, obtained the highest recall of 0.89, outperforming the other machine-learning classifiers. Again, the RF algorithm recorded the highest f1-score compared to the other algorithms.

### 7.3. Evaluation of Machine Learning Models in Segmenting Aerial Satellite Forest Image

**Figure 7:**
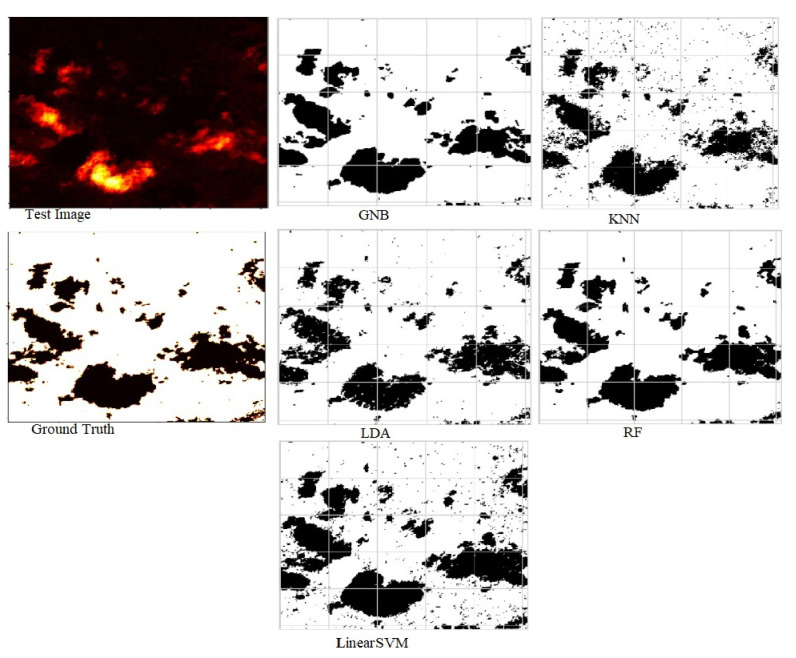
Predicted segmentation results by RF, LDA, GNB, kNN, and LinearSVM with the ensembled approach.

The power of segmenting an aerial forest image for each machine learning was evaluated in terms of RMSE, accuracy, and Jaccard score. As presented in Table 4, the model based on RF outperformed other machine learning segmentation techniques such as Gaussian Naive Bayes (GNB), k Nearest Neighbor (kNN), Linear discriminant analysis (LDA), and Linear Support Vector Machine (LinearSVM) in terms of accuracy, Jaccard score index, and RMSE. In terms of errors, the RF-based model recorded the lowest RMSE of 0.245, and this implies that its predictions are much closer to the actual values than those of other models. Again, the RF-based model also achieved the highest IOU of 0.913, indicating a minimum overlap between the target mask and the predicted output, and also the same technique had the highest accuracy of 0.94 implying that most pixels were classified into their true regions. Figure 7 shows segmented images of the test image with respect to all the classifiers used in a hybrid deep learning approach.

**Figure 8:**
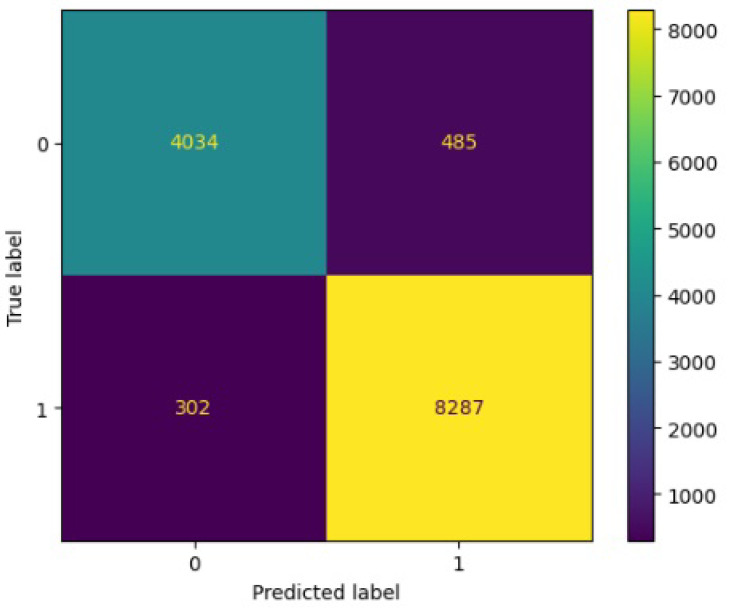
Confusion matrix results for RF.

**Figure 9:**
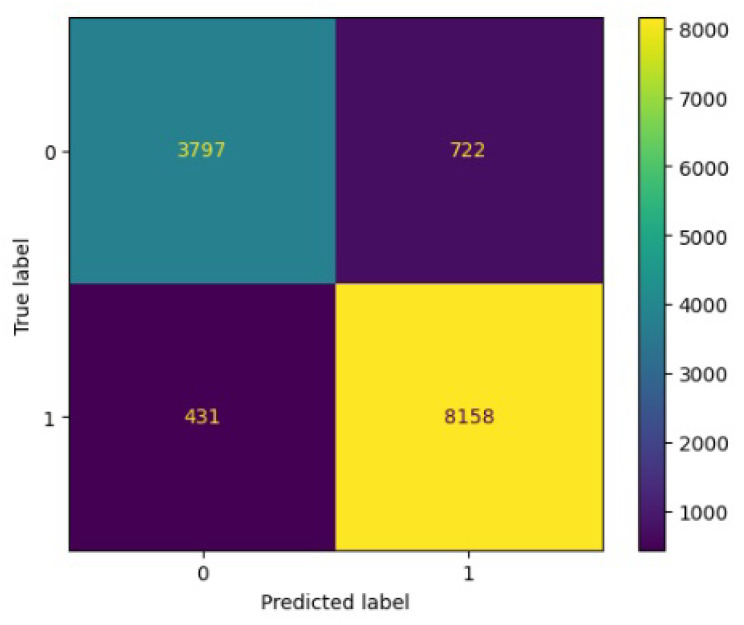
Confusion matrix results for LinearSVM.

**Figure 10:**
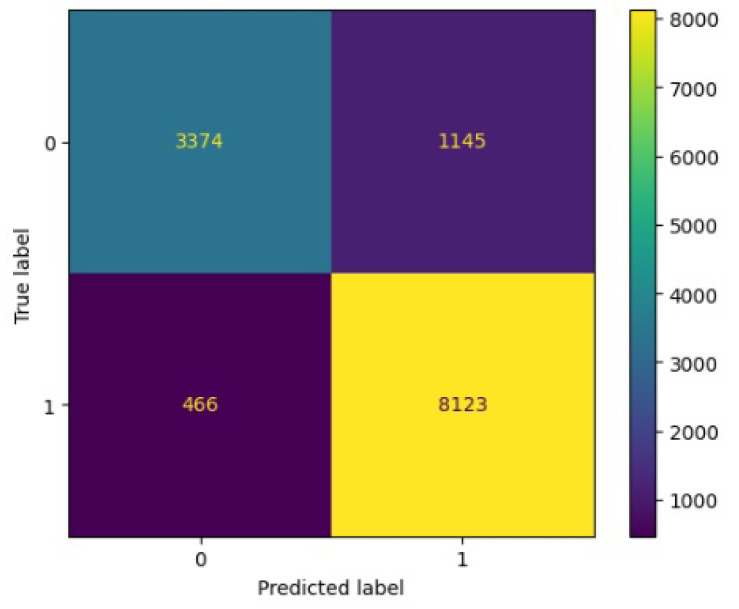
Confusion matrix results for LDA.

**Figure 11:**
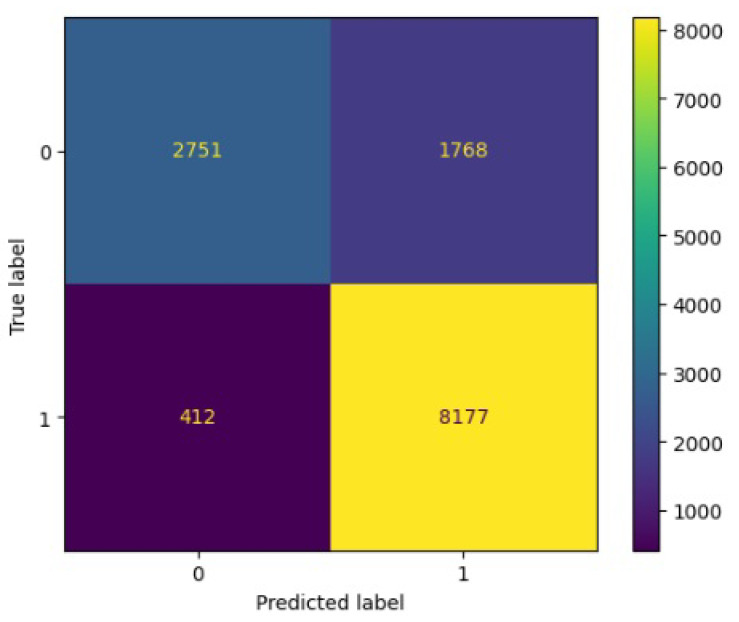
Confusion matrix results for KNN.

**Figure 12:**
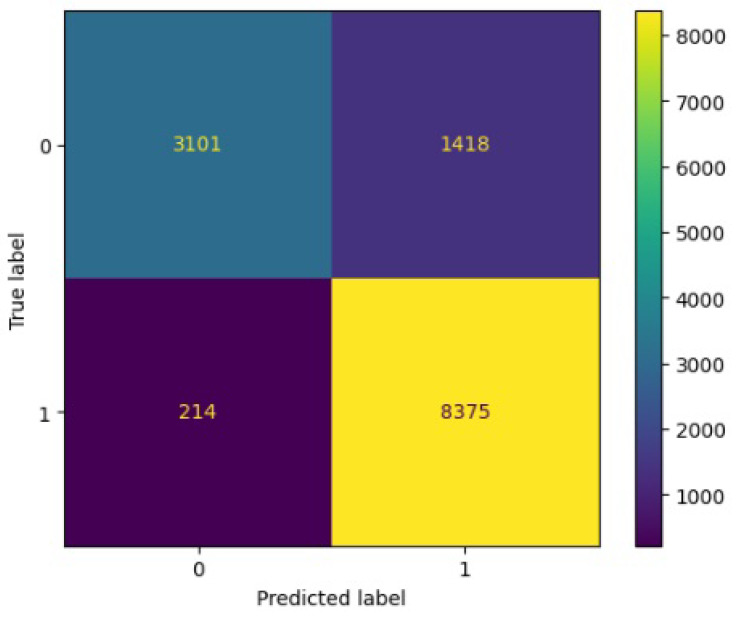
Confusion matrix results for GNB.

**Figure 13:**
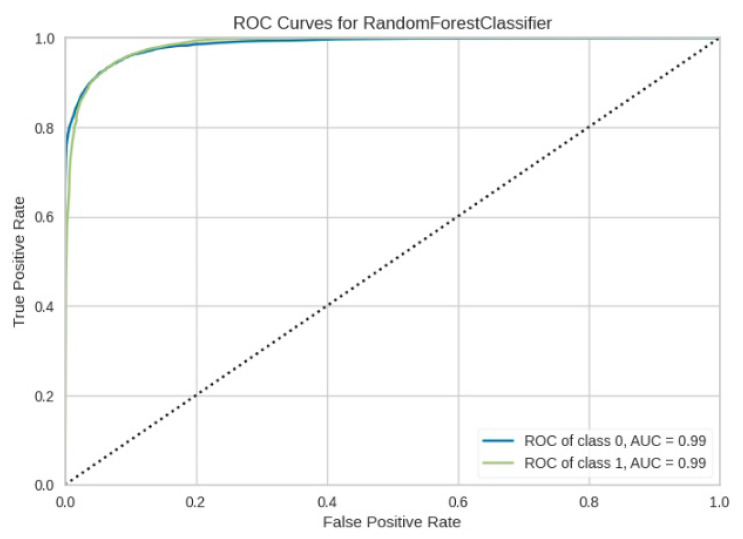
ROC curve for RF.

**Figure 14:**
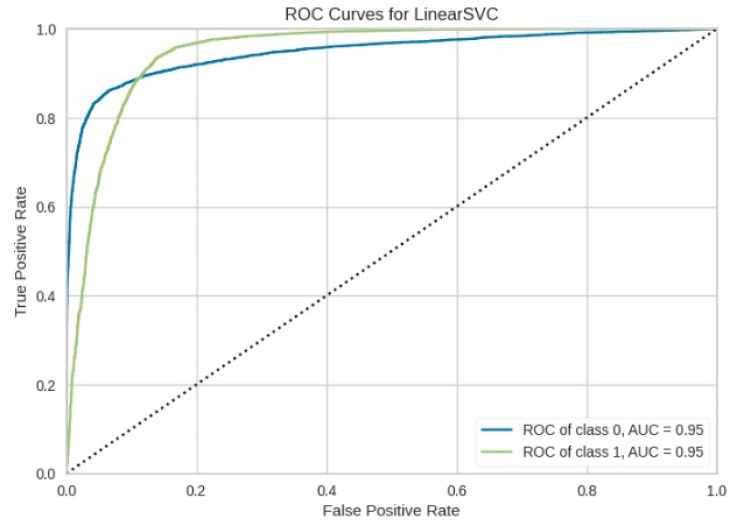
ROC curve for LinearSVM.

**Figure 15:**
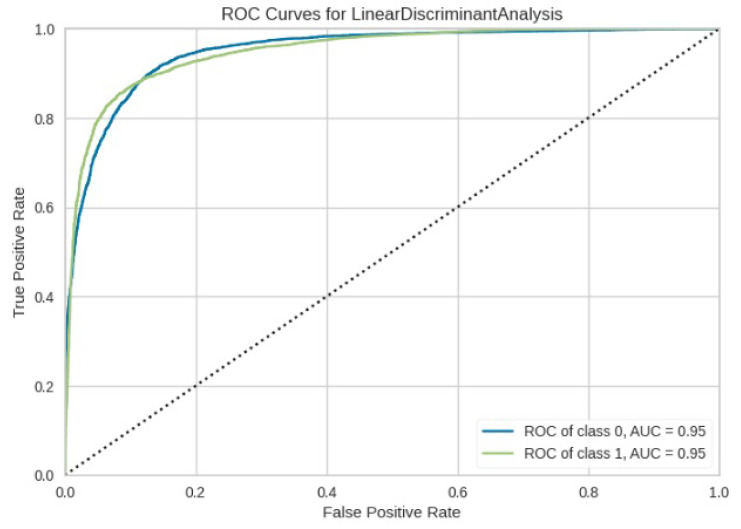
ROC curve for LDA.

**Figure 16:**
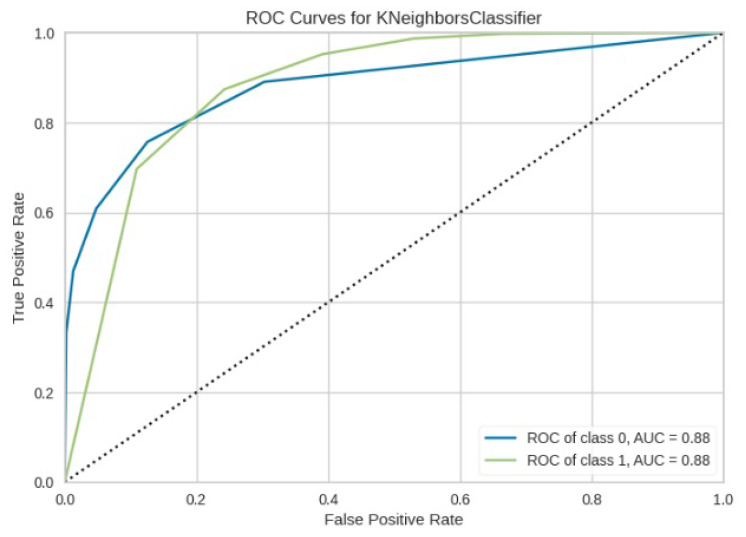
ROC curve for KNN.

**Figure 17:**
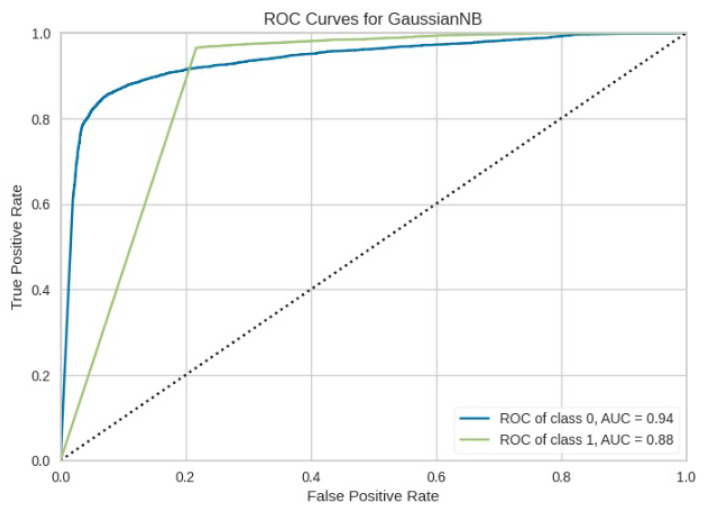
ROC curve for GNB.

Figure 8, Figure 9, Figure 10, Figure 11 and Figure 12 present the confusion matrix of all the classifiers in response to the features obtained from the hybrid approach of deep learning models. The Gaussian Naive Bayes confusion managed to classify 8375 of the 8589 pixels in class 1, while 214 were misclassified as belonging to class 0. Only 1418 pixels were misclassified as class 1 out of 4519 pixels classified as class 0. The model misclassified 1632 pixels in total. The Random Forest-based model recorded the best performance in relation to the other 4 models. The model recorded the least pixel misclassification compared to the other models as it misclassified only 787 pixels. Linear Support Vector Machine confusion matrix indicates that, out of 8589 pixels that belong to class 1, 8123 were correctly classified and 466 were misclassified as belonging to class 0. For 4519 pixels under class 0, 3374 were correctly classified and 1145 were misclassified as class 1. In total, the model misclassified 1611 pixels. The LDA model correctly classified 11,497 pixels and wrongly classified 1611 pixels to other classes. The model based on kNN performed the least of all the other algorithms as it misclassified most pixels into other classes. The ROC_−_AUC curves in Figure 13, Figure 14, Figure 15, Figure 16 and Figure 17 show that all the classifiers have excellent potential to distinguish regions in an image as indicated by their values that are above 0.9. The model based on Random Forest emerged as the best model at distinguishing image objects as it attained the highest ROC_−_AUC value of 0.98.

### 7.4. Evaluation of the Performance of the Classifiers without the Hybrid Deep Learning Approach in Detecting Forest Areas

Table 5 shows the performance of the machine learning classifiers without the hybrid deep learning approaches in detecting forest region areas in terms of precision, recall, and F1 score. The RF, LDA, LinearSVM, and GNB obtained the same precision score of 0.65 with kNN obtaining a slightly low score of 0.64. Again RF, LDA, LinearSVM attained an absolute recall value of 1.0 with kNN performing the least with a recall value of 0.56. Regarding F1-score, kNN performed the least by obtaining an F1 score of 0.49.

### 7.5. Evaluation of the Performance of the Classifiers without the Hybrid Deep Learning Approach in Detecting Non-Forest Areas

Table 6 also shows the performance of the machine learning classifiers without the hybrid deep learning approach in detecting non-forest regions. The RF algorithm had the best precision score of 0.50, while LDA and LinearSVM had the lowest performance with precision values of 0. The kNN approach had the maximum recall value of 0.50, whereas LinearSVM and LDA had the lowest recall values of 0. In terms of F1 score, the kNN machine learning technique outperformed all other classifiers once more, with a value of 0.41.

### 7.6. Evaluation of Machine Learning Models without the Hybrid Deep Learning Approach in Segmenting Aerial Satellite Forest Image

**Figure 18:**
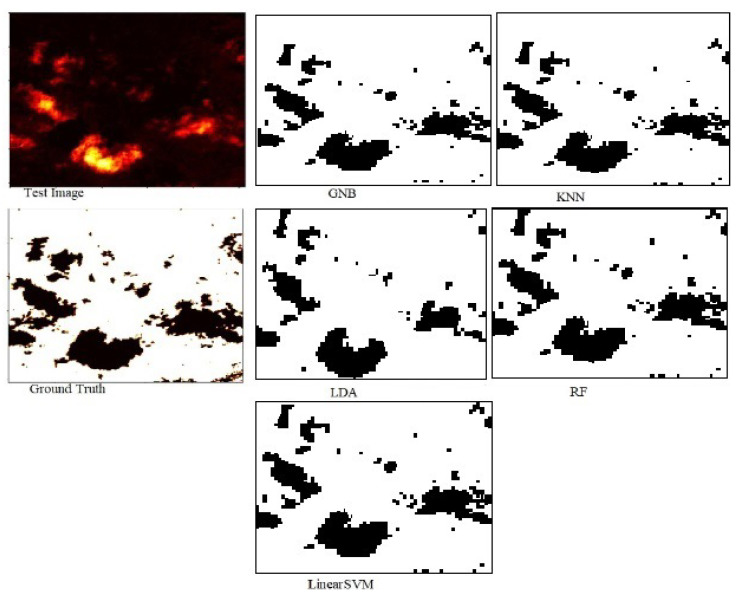
Segmentation of test image with respect to RF, LinearSVM, LDA, GNB, and kNN without the deep learning.

To further evaluate the performance of the five models, we computed the RMSE, Accuracy, and Jaccard score index for each classifier. Table 7 presents the RMSE, Accuracy, and Jaccard score for each classifier. It is shown in Table 7 that the accuracy and Jaccard score of RF, GNB, LDA, and LinearSVM comparatively obtained the same value of around 0.65 which indicates an average performance. The kNN model recorded the highest error of 0.71 in terms of RMSE and overall performed the worst against all other classifiers. Figure 18 shows segmented images of the test image produced by all the classifiers without the deep learning approach.

**Figure 19:**
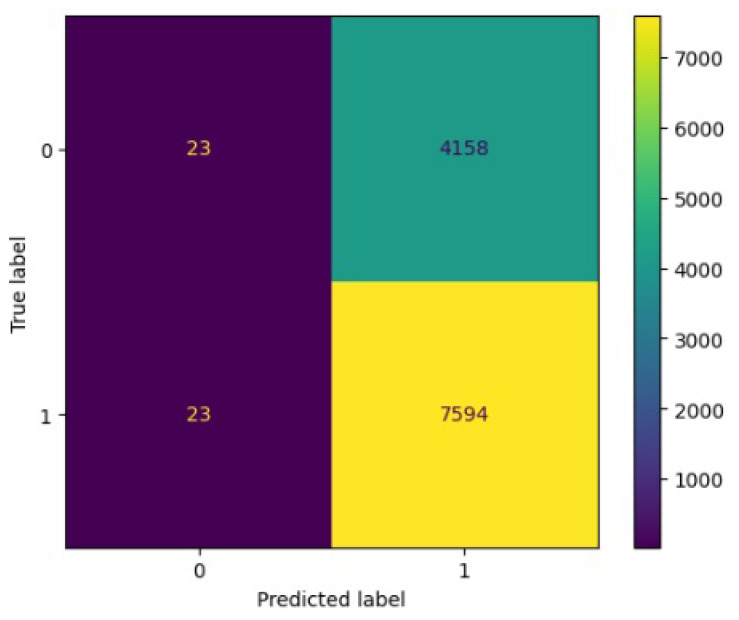
Confusion matrix results for RF without the deep learning.

**Figure 20:**
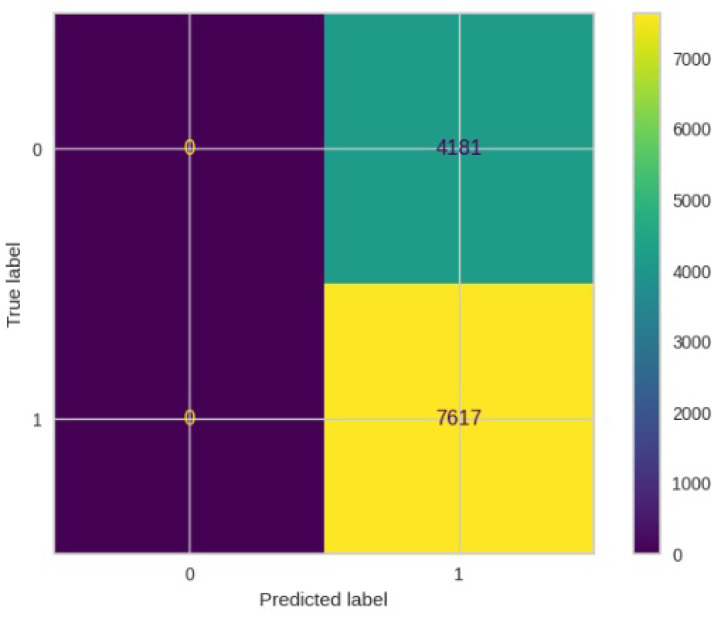
Confusion matrix results for LinearSVM without the deep learning.

**Figure 21:**
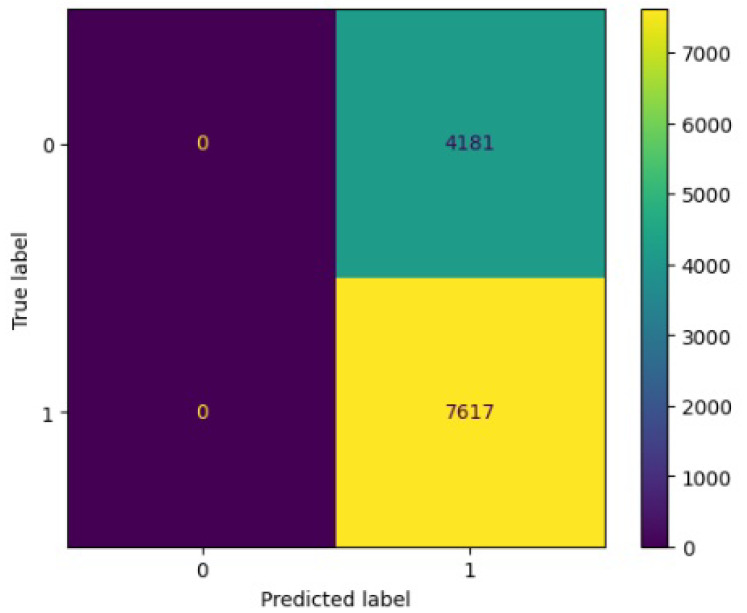
Confusion matrix results for LDA without the deep learning.

**Figure 22:**
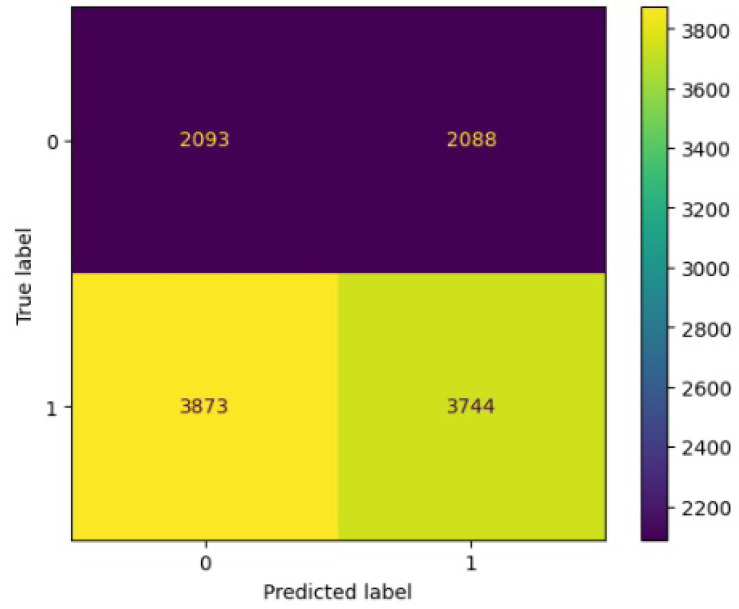
Confusion matrix results for KNN without the deep learning.

**Figure 23:**
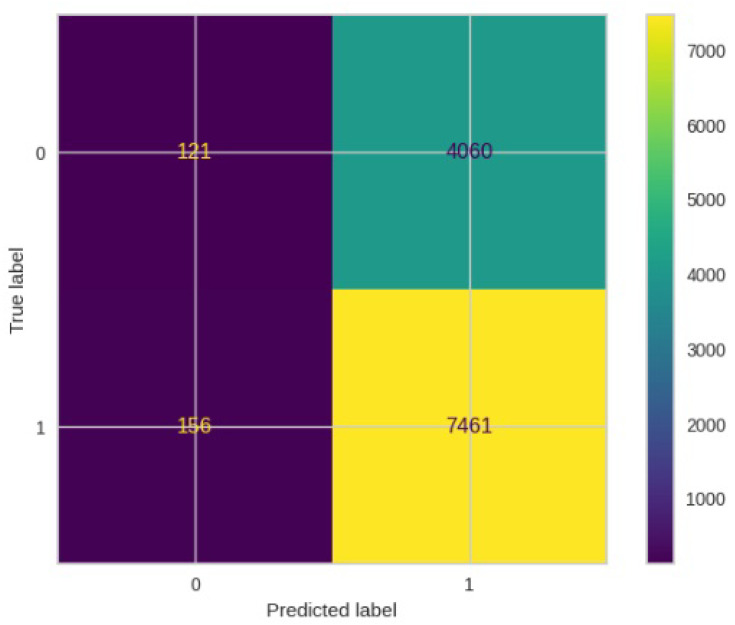
Confusion matrix results for GNB without the deep learning.

Figure 19, Figure 20, Figure 21, Figure 22 and Figure 23 showcase the confusion matrices for all classifiers used individually. In the Gaussian Naive Bayes confusion matrix, 7461 pixels were correctly classified in class 1, while 4060 were erroneously assigned to class 0, resulting in a total of 4216 misclassified pixels. The Random Forest-based model misclassified 4181 pixels in total. The confusion matrix for Linear Support Vector Machine indicates that 7617 pixels were correctly classified, while 4181 were misclassified as belonging to class 0. Similarly, the LDA model exhibited identical performance to the Linear Support Vector Machine. However, the model based on kNN performed the least among all other algorithms, misclassifying the majority of pixels into other classes.

**Figure 24:**
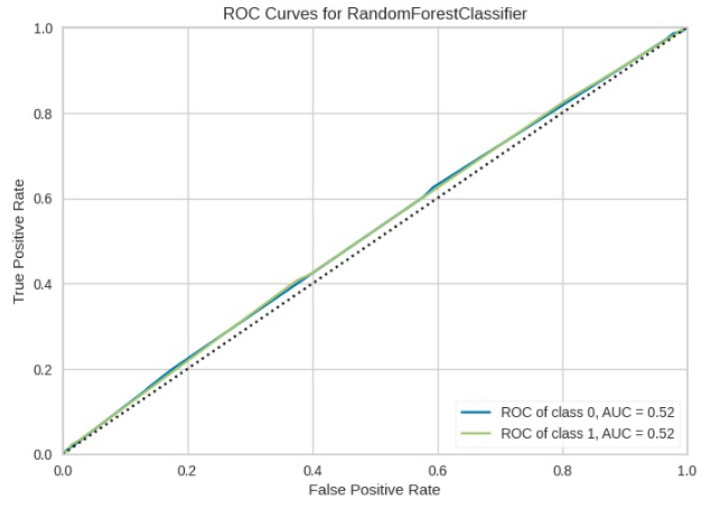
ROC Curve results for RF without the deep learning.

**Figure 25:**
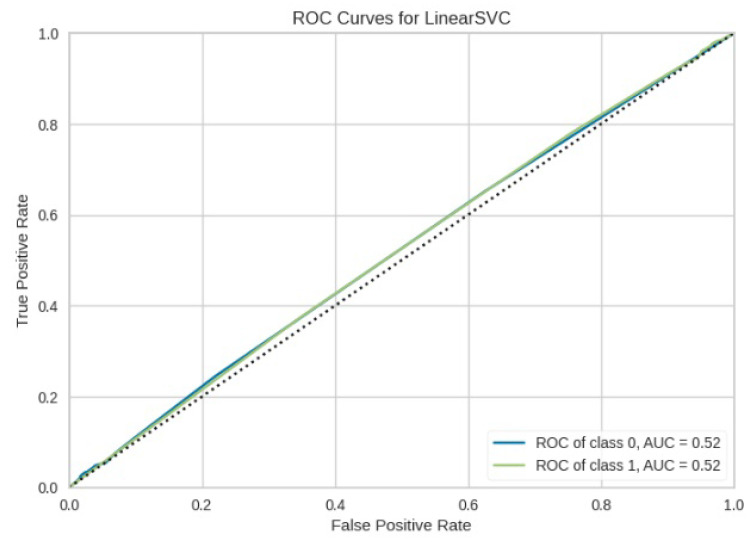
ROC Curve results for LinearSVM without the deep learning.

**Figure 26:**
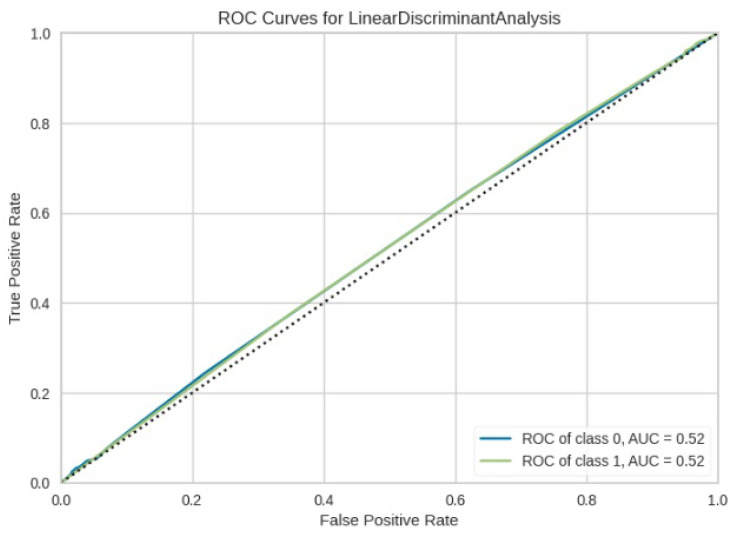
ROC Curve results for LDA without the deep learning.

**Figure 27:**
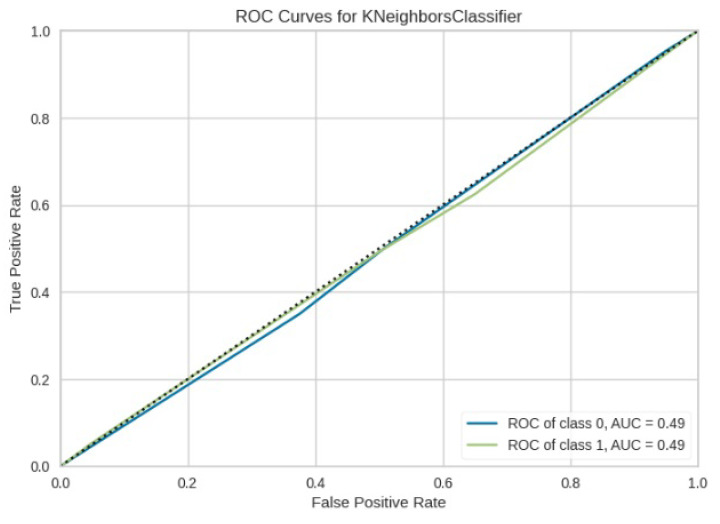
ROC Curve results for KNN without the deep learning.

**Figure 28:**
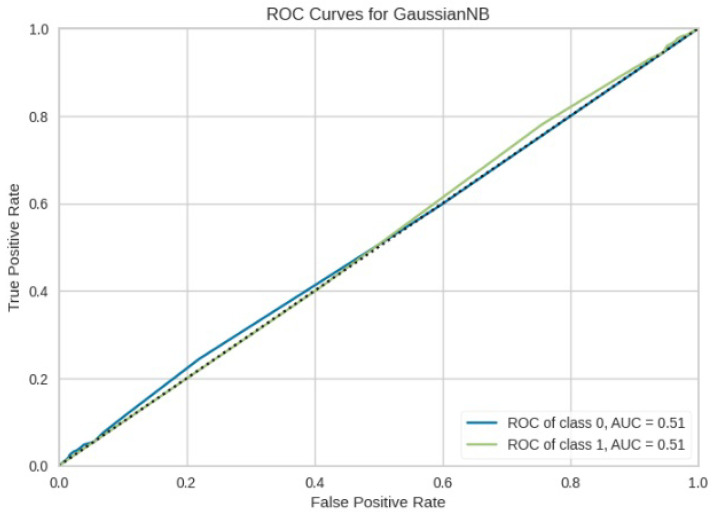
ROC Curve results for GNB without the deep learning.

The ROC_−_AUC values obtained in Figure 24, Figure 25, Figure 26, Figure 27 and Figure 28 indicates that LinearSVM, LDA, GNB, and RF cannot adequately distinguish between image regions because the obtained ROC_−_AUC values lie in between 0.5 to 0.7. A ROC_−_AUC value range between 0.5 to 0.7 means that the model cannot adequately distinguish between image regions range between 0.7 to 0.8 its acceptable discrimination, 0.8 to 0.9 offers good discrimination and values that are greater than 0.9 have excellent discrimination [39]. kNN model alone is recommended to be used in object detection as it obtained a value that is less than 0.5.

### 7.7. Evaluation of the Performance of Deep Learning Models for Detecting Forest and Non-Forest Areas

Table 8 and Table 9 show that the ResNet50 model outperformed the VGG16 model in detecting both forest and non-forest regions.

### 7.8. Evaluating Deep Learning Models in Segmenting Aerial Satellite Image

**Figure 29:**
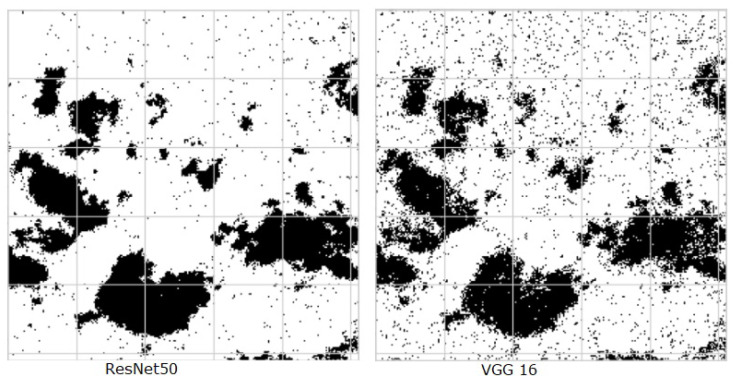
Predicted segmentation results by ResNet50 and VGG16 models.

The performance of deep learning models was assessed using RMSE, Accuracy, and Jaccard score index. According to Table 10, the ResNet50 model achieved an accuracy of 0.91, an RMSE of 0.29, and a Jaccard score of 0.87. In comparison, the VGG16 model obtained an accuracy of 0.85, an RMSE of 0.37, and a Jaccard score of 0.80. Results obtained show that ResNet50 outperforms VGG16 across all evaluated metrics. ResNet50 achieved higher accuracy (0.91 vs. 0.85), lower RMSE (0.29 vs. 0.37), and a higher Jaccard score (0.87 vs. 0.80) compared to VGG16. This indicates that ResNet50 is more accurate, has lower prediction error, and has better overlap between predicted and actual values than VGG16. Figure 29 shows segmented images by ResNet50 and VGG16 models.

**Figure 30:**
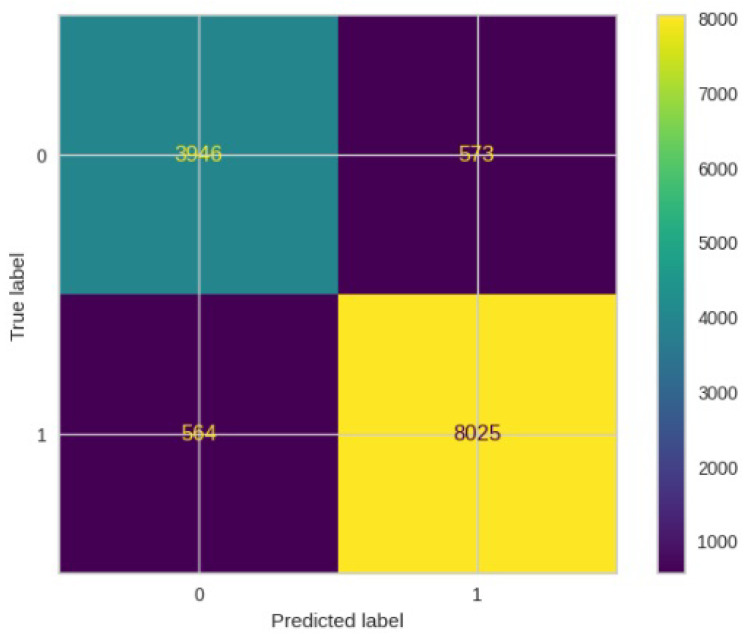
Confusion matrics for ResNet50.

In Figure 30, the confusion matrix depicts the performance of the ResNet50 model. It correctly classified 8025 out of 8589 pixels in class 1, but misclassified 564 pixels as belonging to class 0. Out of 13108 pixels in total, 564 were erroneously classified into class 0, while 573 were mistakenly assigned to class 1.

**Figure 31:**
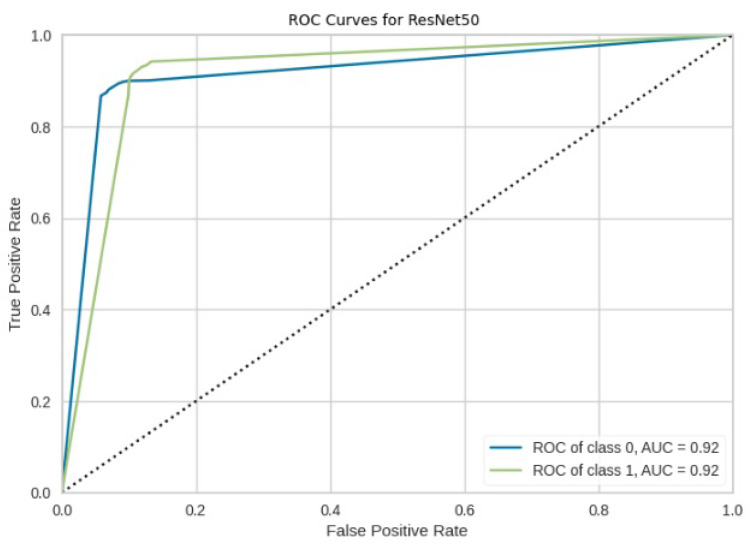
ROC curves for ResNet50.

The ROC-AUC curve displayed in Figure 31 for ResNet50 indicates that the model effectively discriminates between forest and non-forest regions, achieving a good ROC-AUC value of 0.92.

**Figure 32:**
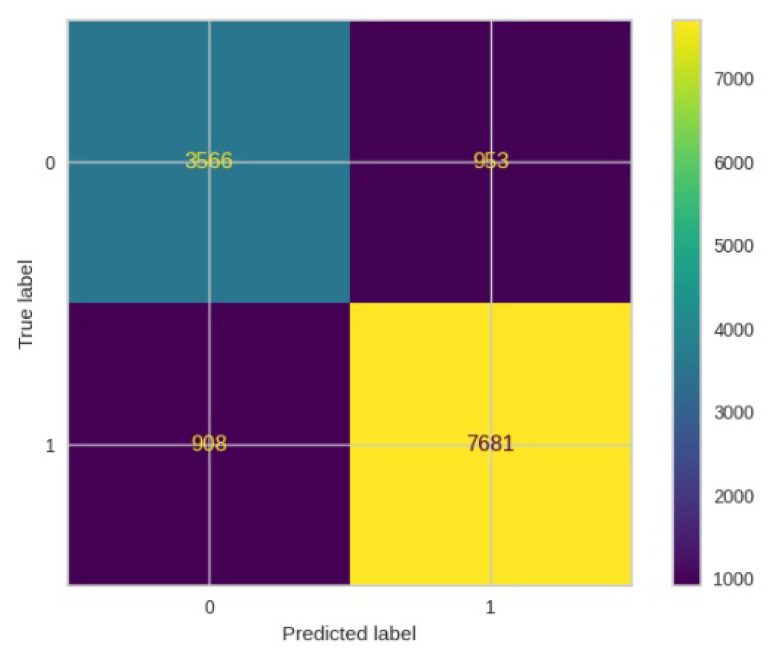
Confusion matrics for VGG16.

In Figure 32, the confusion matrix depicts the performance of the ResNet50 model. It correctly classified 7681 out of 8589 pixels in class 1, but misclassified 908 pixels as belonging to class 0. Out of 13108 pixels in total, 908 were mistakenly categorized as class 0, while 953 were misclassified as class 1.

**Figure 33:**
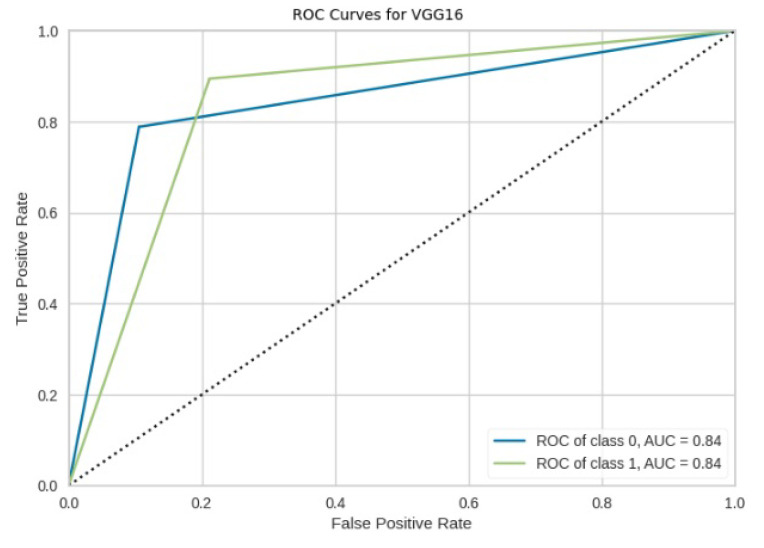
ROC curves for VGG16.

The ROC-AUC curve displayed in Figure 33 for ResNet50 indicates that the model effectively discriminates between forest and non-forest regions, achieving a commendable ROC-AUC value of 0.84.

Table 11 shows a comparison in performance between the machine learning classifiers with the hybrid deep learning approach, the classifiers alone, and the deep learning models in terms of accuracy, RMSE, and Jaccard index score. Both the hybrid deep learning approach and the deep learning models significantly outperformed the classifiers alone across all metrics. This is attributed to the fact that the classifiers alone do not have the capacity to extract features such as spatial relationships between pixel and texture. Therefore classifiers should be used in a pipelined fashion where they perform the process of segmentation after receiving features from other models. This is the reason why the classifiers used in conjunction with deep learning hybrid approaches produced satisfactory results. In the hybridization approach, the RF algorithm emerged as the winner in performing the segmentation task. These results also go in glove with results obtained by [40] where the Random Forest approach outperformed other algorithms such as Gentle AdaBoost (GAB), Maximum Likelihood Classification (MLC), and Support Vector Machines (SVM) in a pipelined approach fashion. Another study by [41] demonstrated that the RF algorithm performs well in object detection for multi-spectral images.

It is important to evaluate segmentation algorithms to determine algorithms suitable for a given application. Algorithm performance is dependent on the type of images used. Images are generally classified into, synthetic, remote sensing, medical and natural images. A particular algorithm might be better in remote sensing images but poor in medical images. In light of the evaluation of the algorithms, the Random Forest algorithm outperformed Linear Discriminant Analysis, Gaussian Naive Bayes, and Support vector machines algorithms in terms of accuracy, Jaccard score, and ROC curves. As presented in Table 12 the proposed model in this study outperformed other models from related studies [10,42,43,44,45,46]. However, the Unet semantic segmentation in [47] segmenting the forest images and predicting any loss (deforestation) or gain (reforestation) slightly outperformed our model with 95% accuracy. The reason could be attributed to the ability of Unet to extract more features required to perform subsequent segmentation.

## 8. Conclusions

This paper adopts a hybridized approach of deep learning models and traditional machine learning classifiers used to identify forest and non-forest areas from an aerial satellite image obtained from the Deep Globe challenge dataset. A deep learning hybrid approach of VGG16 and ResNet50 was used to extract a set of features that were subsequently used by machine learning classifiers to segment an aerial satellite image into the forest and non-forest areas. Metrics such as IoU, accuracy, RMSE, and ROC_−_AUC curves were used to assess the performance of the models. The model based on RF emerged as the winner as it achieved an accuracy of 94% and an IoU of 91%. The high efficacy of the model implies that the model can be used to detect smoke, veld fires, and perform water segmentation. The ensemble edge vector approach contributed to the high efficacy of the model. The opaque nature of deep learning models, stemming from their complex network structures, renders them as black boxes, making it challenging to comprehend their decision-making processes [49]. Consequently, domain experts may struggle to ascertain whether these models have accurately acquired the relevant knowledge, potentially eroding users’ trust in deep learning systems. These models have a huge limit in working with symbolic information. This limitation presents a significant obstacle for end users of Earth Observation applications who are accustomed to working with symbolic information, such as ecologists, agronomists, and other related professionals [50]. Therefore, The future of remote sensing science should be supported by knowledge representation techniques such as ontologies [49]. For future work, it is recommended to include more classes and to adopt high-resolution networks (HRnets) as an alternative to VGG16 and ResNet50 because of their ability to perform low-resolution to high-resolution conversion, which is also linked to their block network architectures constructed according to new standards, and therefore excels at vision tasks such as feature extraction and object detection.

## Figures and Tables

**Table 1:** Hardware and Software specification for the experiment.

Hardware	Software
Processor: core i5 2.2 Gigahertz	Programming language: Python version 3.9
RAM: 32 Gigabytes	Backend: TensorFlow GPU
Graphical Processing Unit (GPU)	DeepLearning API: Keras GPU
Harddrive: 500 Gigabytes	
NVIDIA, 16 Gigabytes RAM	

**Table 2:** Metrics of classifiers in terms of precision, recall, and F1-Score with a hybrid approach of deep learning for detecting forest areas.

Metric	kNN	RF	LDA	LinearSVM	GNB
Precision	0.82	0.94	0.88	0.92	0.86
Recall	0.95	0.96	0.95	0.95	0.98
F1-Score score	0.88	0.94	0.88	0.91	0.88

**Table 3:** Metrics of classifiers in terms of precision, recall, and F1-Score with a hybrid approach of deep learning for detecting non-forest areas.

Metric	kNN	RF	LDA	LinearSVM	GNB
Precision	0.87	0.93	0.88	0.90	0.94
Recall	0.61	0.89	0.75	0.84	0.69
F1-Score score	0.72	0.91	0.81	0.87	0.79

**Table 4:** Metrics of classifiers in terms of accuracy, RMSE, and Jaccard score with a hybrid approach of deep learning in segmenting aerial satellite forest image.

Metric	kNN	RF	LDA	LinearSVM	GNB
RMSE	0.408	0.245	0.351	0.332	0.353
Accuracy	0.833	0.940	0.877	0.890	0.876
Jaccard score	0.790	0.913	0.834	0.876	0.837

**Table 5:** Metrics of classifiers in terms of precision, recall, and F1-Score without the deep learning hybrid approach in detecting forest areas.

Metric	kNN	RF	LDA	LinearSVM	GNB
Precision	0.64	0.65	0.65	0.65	0.65
Recall	0.49	1.00	1.00	1.00	0.98
F1-Score score	0.56	0.78	0.78	0.78	0.78

**Table 6:** Metrics of classifiers in terms of precision, recall, and F1-Score without the deep learning hybrid approach in detecting non-forest areas.

Metric	kNN	RF	LDA	LinearSVM	GNB
Precision	0.35	0.50	0.00	0.00	0.44
Recall	0.50	0.01	0.00	0.00	0.03
F1-Score score	0.41	0.01	0.00	0.00	0.05

**Table 7:** Metrics of classifiers in terms of accuracy, RMSE, and Jaccard score without the hybrid approach of deep learning in segmenting aerial satellite forest image.

Metric	kNN	RF	LDA	LinearSVM	GNB
RMSE	0.711	0.595	0.595	0.595	0.598
Accuracy	0.495	0.646	0.646	0.645	0.643
Jaccard score	0.386	0.645	0.646	0.646	0.639

**Table 8:** Evaluating Deep learning models in terms of precision, recall, and F1-Score in detecting non-forest areas.

Metric	ResNet50	VGG16
Precision	0.88	0.80
Recall	0.88	0.79
F1-Score score	0.88	0.79

**Table 9:** Evaluating Deep learning models in terms of precision, recall, and F1-Score in detecting forest areas.

Metric	ResNet50	VGG16
Precision	0.94	0.89
Recall	0.93	0.89
F1-Score score	0.93	0.89

**Table 10:** Evaluating deep learning models in terms of accuracy, RMSE, and Jaccard score in segmenting aerial satellite forest image.

Metric	ResNET50	VGG16
RMSE	0.29	0.38
Accuracy	0.91	0.85
Jaccard score	0.88	0.80

**Table 11:** Performance comparison of classifiers with hybrid deep learning approach, classifiers alone, and deep learning models in terms of RMSE, Accuracy, and Jaccard score index.

Classifiers with Hybrid Deep Learning Approach						Classifiers Alone					Deep Learning Models	
**Metric**	**RF**	**LinearSVM**	**LDA**	**GNB**	**kNN**	**RF**	**LinearSVM**	**LDA**	**GNB**	**kNN**	**ResNet50**	**VGG16**
Accuracy	0.940	0.890	0.877	0.876	0.833	0.646	0.646	0.646	0.643	0.49	0.913	0.852
Jaccard Score	0.913	0.876	0.834	0.837	0.790	0.645	0.646	0.646	0.639	0.386	0.876	0.804
RMSE	0.245	0.332	0.351	0.535	0.408	0.595	0.594	0.595	0.600	0.711	0.295	0.377

**Table 12:** Accuracy and IOU obtained from other studies.

Method	Accuracy	IOU
Unet with spatial pyramid spooling [42]	86.71	75.59
Hnet with Inception as backbone [43]	68	83
Deep Convolutional Neural Networks (DCNN) [48]	91	-
Unet for forest segmentation [10]	91	-
SENet and MobileNet embedded in DeepLabV3+ (SMED) [44]	82.95	60
improved tuna swarm optimization (ITSO) [46]	-	59
Unet semantic segmentation [47]	95	-
Random Forest	94	91

## Data Availability

The data that support the findings of this study are available on [36]. The authors confirm that the data supporting the findings of this study are available within the article.
